# Alanine-based spacers promote an efficient antigen processing and presentation in neoantigen polypeptide vaccines

**DOI:** 10.1007/s00262-023-03409-3

**Published:** 2023-02-23

**Authors:** Carmen Aguilar-Gurrieri, Ana Barajas, Carla Rovirosa, Raquel Ortiz, Victor Urrea, Nuria de la Iglesia, Bonaventura Clotet, Julià Blanco, Jorge Carrillo

**Affiliations:** 1 IrsiCaixa AIDS Research Institute, Germans Trias I Pujol Research Institute (IGTP), Badalona, Spain; 2 University of Vic-Central University of Catalonia (UVic-UCC), Vic, Spain; 3 Infectious Diseases Department, Germans Trias I Pujol Hospital, Badalona, Spain; 4 CIBER de Enfermedades Infecciosas, Madrid, Spain

**Keywords:** Neoantigen, Cancer vaccine, Immunotherapy, Antigen processing, Antigen presentation

## Abstract

Neoantigens are tumor-specific antigens that are mostly particular for each patient. Since the immune system is able to mount a specific immune response against these neoantigens, they are a promising tool for the development of therapeutic personalized cancer vaccines. Neoantigens must be presented to T cells by antigen presenting cells (APC) in the context of MHC-I or MHC-II molecules. Therefore, the strategy of vaccine delivery may have a major impact on the magnitude and quality of T cell responses. Neoantigen-based vaccines are frequently administered as a pool of individual synthetic peptides that induce mainly CD4^+^ T cell responses. MHC-I-mediated presentation and the elicitation of CD8^+^ T cell responses may be improved using DNA or RNA sequences that code for a unique long polypeptide that concatenates the different neoantigens spaced by linker sequences. When administered this way, the selection of the spacer between neoantigens is of special interest, as it might influence the processing and presentation of the right peptides by APCs. Here, we evaluate the impact of such linker regions on the MHC-I-dependent antigen presentation using an in vitro assay that assesses the MHC-I presentation of SIINFEKL, a H-2 Kb-restricted OVA peptide. Our results show that spacers used to generate epitope concatenates have a large impact on the efficiency of neoantigen processing and presentation by MHC-I molecules; in contrast, the peptide position and the flanking regions have a minimal impact. Moreover, linkers based on alanine residues promote a more efficient peptide presentation than the commonly used GGGS linker.

**Supplementary Information:**

The online version contains supplementary material available at 10.1007/s00262-023-03409-3.

## Introduction

Tumoral cells contain a large number of tumor antigens that might be the target of new cancer immunotherapies. Such antigens can be categorized as tumor-associated antigens (TAA) or tumor-specific antigens (TSA) [1]. While TAAs may also be present in non-tumoral cells, thereby being subject to tolerance mechanisms that limit the antitumor immune response [1, 2], TSAs may be considered as neoantigens. TSAs may derive from either oncogenic viral antigens, individualized somatic mutations or altered gene expression (including alternative splicing and frame shifts) and are usually exclusive to each cancer patient (1,2). Neoantigens are not present in normal tissues, thereby avoiding immune tolerance and playing a major role in T cell-mediated antitumor immunity [3, 4]. Therefore, personalized neoantigen vaccines, targeting multiple neoepitopes, represent a potential new class of cancer immunotherapy. In that sense, a number of promising pre-clinical assays in mice and clinical trials in humans have been conducted using different neoantigen vaccination strategies, including: (i) personalized RNA mutanome vaccines [4, 5], (ii) DNA vaccines [6], (iii) antigen-loaded dendritic cell vaccine [7] and (iv) synthetic long peptides [8, 9].

The success of a cancer vaccine based on neoantigens relies on four components: immunogenic neoantigens, formulations, immune adjuvants and delivery vehicles [1, 10]. Neoantigens can be presented to the immune system as a pool of short (8 to 11 amino acids) or long (15 or more residues) individual synthetic peptides or as a unique long single molecule that concatenates the different neoantigens spaced by linker sequences (prepared as DNA, RNA or soluble protein). Short peptides (SP) can bind exogenously to the MHC-I on the cell surface, while long peptides (LP) need to be internalized and efficiently processed into both the MHC-I and -II antigen presentation pathways by APCs [11]. Both SPs and LPs need to be administered in combination with potent adjuvants in order to overcome their low immunogenicity [12, 13]. Unlike SPs, which mainly induce CD8^+^ T cell responses, LPs have been shown to generate both CD8^+^ and CD4^+^ T cell responses, broadening the immune cellular response [11, 14, 15]. This may represent a clear advantage with respect to short peptides, since CD4^+^ T cells play a key role in orchestrating the immune response and are crucial to fully activate CD8^+^ T cells. However, and at the same time, this could be considered as a drawback since they can promote the generation of CD4^+^ T cells more efficiently than CTL responses, despite neoantigens were selected based on MHC-I presentation [6, 9]. In addition, during the trimming process, LP might generate a mix of useless short peptides that can affect to the presentation of the neoantigen of interest. To avoid that endogenous neoantigen flanking sequences may be replaced by specific designed ones that promote the processing and presentation of the selected neoantigen.

Short and long peptides may be concatenated in a longer polypeptide sequence containing multiple selected neoepitopes, expanding the potential efficacy of neoantigen vaccines. Such polypeptide should be designed in a way that individual neoantigens can be properly presented by MHC molecules after being processed by the antigen presentation machinery. In the case of the MHC-I antigen presentation route, individual peptides are generated in the cytosol by the immunoproteasome [16, 17], transported to the endoplasmic reticulum by TAPs, and finally trimmed by the ERAP1/2 proteins [18–20] and loaded onto the MHC-I molecules [21]. Therefore, since flanking sequences are playing a major role in MHC-I antigen presentation, special care needs to be taken in the selection of spacers flanking each neoantigen sequence, in order to enhance the correct processing and presentation of intact neoepitopes. No clear consensus is found in the literature about which type of linker would be optimal, as a variety of linkers has been used so far, including the naturally flanking residues of the neoantigen [6, 22–31]. The right choice of spacer is key for the design of concatenated neoantigen-based vaccines delivered as DNA, RNA or soluble polypeptide. Here, we have evaluated the impact that the linkers may have on MHC-I presentation of neoantigens included in a polypeptide vaccine using a surrogate peptide. In addition, we selected linker sequences that enhance proper peptide processing and presentation on MHC-I molecules.

## Materials and methods

*Cell line*. B16-F10 cells (ATCC CRL-6475) were grown and maintained in Dulbecco’s Modified Eagle Medium (DMEM containing 4.5 g/L D-/L-Glucose and 110 mg/L Sodium Pyruvate) (Gibco) supplemented with 10% heat-inactivated fetal bovine serum (FBS) (Gibco). These cells were used for all the experiments hereby.

*Design and molecular cloning of multi-epitope genes*. Thirteen B16-F10-derived neoantigens (nine amino acids long) were selected based on their putative binding to MHC-I. We designed a single chimeric gene containing (from N-terminal to C-terminal ends): a signal peptide (MDWTWRFLFVVAAATGVQS), a FLAG tag (DYKDDDDK), all selected peptides, the OVA peptide (SIINFEKL) at several position depending on the construct, and the CD44 transmembrane domain (cytosolic domain of CD44 was not included). Thus, the recombinant proteins were designed to be detected on the surface of the transfected B16-F10 cells by flow cytometry (Supplementary Fig. 1). All regions and peptides were joined using the following specific short linker: 1) AAA (5’-GCTGCTGCC-3’), 2) AAL (5´-GCTGCCCTG-3´), 3) ADL (5´-GCTGACCTG-3´), 4) A (5´-GCC-3´) and 5) GGGS (5´-GGAGGCGGCTCT-3´). One individual construction was prepared for each linker used. In addition, an intracellularly expressed version of each polypeptide described above were designed lacking the signal peptide and the CD44 transmembrane domain. These artificial genes were synthetized by GeneArt (Invitrogen) and cloned in a pcDNA3.4 vector (Invitrogen).

*Expression and western blot analysis.* B16-F10 cells were seeded in a 12 well-plate at a rate of 1 × 10^5^ cells/well. Cells were transfected 24-h post-seeding using the Lipofectamine 3000 Reagent (Thermo Fisher Scientific). Six-hour post-transfection, cells were stimulated with mouse IFN-γ (Biolegend) at a final concentration of 20 ng/mL and incubated for 48 h. After that, cells were incubated with 10 µM of MG132 (Sigma) for 4 h. Only stimulated and only treated cells, as well as untreated and unstimulated cells, were also included in the experiment as controls. Cells were washed twice with 1xPBS pH 7.4 (Gibco), detached using Versene (Gibco), transferred to a clean tube and centrifuged for 5 min at 420xg. Cell pellet was resuspended in lysis buffer containing 1 × RIPA buffer (Cell Signaling) and protease inhibitors (HALT (Thermo Scientific) and PMSF (Cell Signaling)), and centrifuged for 10 min at 10,000 rpm. Supernatant containing the cell lysate was transferred to a new tube, and proteins were discriminated by SDS-PAGE using 4–12% Bis–Tris Nu-PAGE (Thermo Fisher Scientific) and electro-transferred to a PVDF membrane using the Trans-Blot Turbo Transfer Pack (BioRad). Nonspecific binding sites were blocked using 1xPBS pH 7.4, 0.05% of Tween20, and 5% of non-fat skim milk (blocking buffer) at room temperature (RT) for one hour. Subsequently, the membrane was washed 3 times with wash buffer (1 × PBS, 0.05% Tween20), and incubated with diluted mouse anti-FLAG antibody (MA1-91,878) (Thermo Fisher Scientific) (1:1000) with gentle shaking overnight at 4ºC. Alternatively, the membrane was incubated with diluted anti beta-actin monoclonal antibody-HRP conjugated (clone BA3R, Thermo Fisher Scientific) (1:3000) with gentle shaking for 1 h at RT. The washed membrane was incubated with diluted Peroxidase AffiniPure Donkey anti-mouse IgG (H + L) antibody (Jackson ImmunoResearch) (1:10,000) with gentle shaking for 1 h at RT. Finally, the membrane was developed using the SuperSignal West Pico PLUS Chemiluminescence Substrate (Thermo Fisher Scientific), and images were obtained using a Chemidoc^TM^MP Imaging System (BioRad).

*Expression and flow cytometry analysis.* B16-F10 cells were transfected as indicated above. Six-hour post-transfection, cells were stimulated with mouse IFN-γ (Biolegend) at a final concentration of 20 ng/mL and incubated for 48 h. After that, cells were incubated with 10 µM of MG132 for 4 h. Untreated and unstimulated cells were used as controls. To detach cells, they were washed twice with 1xPBS pH7.4 (Gibco) and incubated with Versene (Gibco). Detached cells were stained extracellularly using APC anti-Flag antibody (clone L5, Biolegend) (1:400). For some experiments, cells were fixed and permeabilized using the Fix&Perm Kit (Invitrogen), and anti-Flag staining was performed intracellularly. After staining, cells were washed, fixed in formaldehyde 1% and acquired in a BD FACSCelesta flow cytometer. Data were analyzed using the Flow-Jo v10.6.2 Software (Tree Star Inc.).

*Epitope presentation assay.* B16-F10 cells were transfected as indicated above or using a mix of two plasmids in a 1:4 ratio. 1) pMAX GFP vector (Amaxa), a GFP expressing control plasmid, and 2) pcDNA3.4 coding for the polypeptide. Six-hour post-transfection, cells were stimulated with mouse IFN-γ (Biolegend) at a final concentration of 20 ng/mL and incubated for 48 h. As positive control, non-transfected but IFN-γ stimulated cells were incubated with the OVA peptide (SIINFEKL) (InvivoGen) at a final concentration of 10 µM for 2 h prior to cell harvest as indicated before. Cells were centrifuged at 420xg for 5 min, resuspended in 1xPBS and stained using APC-conjugated anti-mouse H-2K^b^D^b^ (clone 28–8-6, Biolegend) (1:1000) and the PE-or APC-conjugated anti-mouse H-2 Kb/SIINFEKL (clone 25-D1.16, Biolegend) (1:500 both). Cells were washed, fixed in formaldehyde 1% and acquired in a FACSCelesta flow cytometer (BD Biosciences). Acquired data were analyzed using the Flow-Jo software (Tree Star Inc.).

*Splenocyte activation and proliferation assay.* B16-F10 cells were transfected as indicated above. On day 2 post-transfection, cells were detached with Versene (Gibco) and seeded at a density of 10.000 cells per well in flat bottom 96-well plates (a 1:20 ratio with splenocytes). Splenocytes from OT-I mice (Charles River) were stained with CFSE (0.25 µM) for 5 min at RT. After that, R10 medium was added, and cells were collected by centrifugation and thoroughly washed with 1xPBS (Gibco) at least three times. Cells were resuspended in R10 medium at 2 M/ml. 2 × 10^5^ splenocytes/well were added to the previously seeded plate with the transfected B16-F10. As a positive controls, the OVA peptide (InvivoGen) (1 ng/mL) and Concanavalin A (L7647-25MG, Merck) (2 µg/ml) were used. Finally, an anti-CD28 antibody (16–0281-82, Thermo Fisher) was added at 1 µg/mL for co-stimulation. Activation and proliferation of CD8^+^ OT-I cells were analyzed at 24 and 72 h, respectively, by flow cytometry. In brief, cells were harvested and stained with viability stain solution (565,388, BD) at a 1:4000 dilution and were incubated for 15 min at RT. After washing three times, cells were stained with the following antibodies: anti-CD19 (560,245, BD), anti-CD3e (551,163, BD), anti-CD4 (56–0042-82, Thermo Fisher), anti-CD8 (560,778, BD), anti-CD25 (566,228, BD) and anti-CD44 (561,862, BD). Finally, cells were washed, fixed with a formaldehyde 1% solution and acquired in a FACSCelesta flow cytometer (BD Biosciences). Acquired data were analyzed using the Flow-Jo software (Tree Star Inc.).

*Statistical analysis*. Levels of MHC-I/SIINFEKL on the surface of B16F10 cells were expressed as geometric mean (GeoMean) and percentage of positive cells. Data were analyzed using a Kruskal–Wallis test corrected for multiple comparisons using the Dunn’s test or by False Discovery Rate (FDR) method of Benjamini and Hochberg in the GraphPad Prism 7.0e software. The exact test used in each experiment is indicated at the corresponding figure caption.

## Results

### Development of an epitope presentation assay

**Fig. 1:**
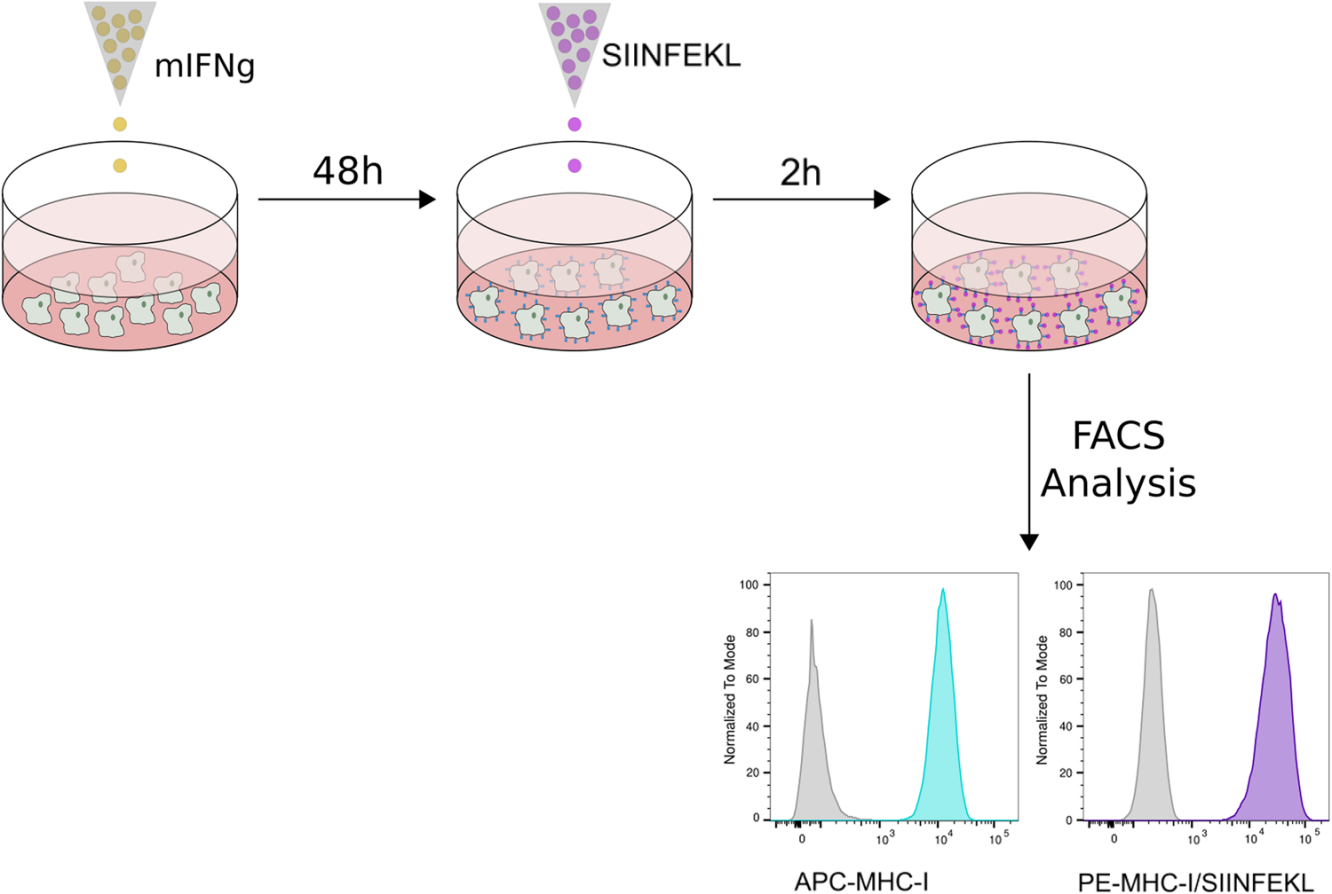
Experimental approach overview. B16-F10 cells were stimulated with INF-γ for 48 h. After that the SIINFEKL peptide was added. The expression of MHC-I and the presence of MHC-I/SIINFEKL complexes were analyzed by flow cytometry. Gray histogram: unstimulated B16-F10 cells; light blue histogram: MHC-I expression in IFN-γ treated cells; purple histogram: MHC-I/SIINFEKL on the surface of cells stimulated with IFN-γ  + SIINFEKL peptide6

To estimate the impact of the spacer sequence on MHC-I antigen presentation, we have developed an in vitro assay that evaluates the presentation of the H-2 Kb-restricted OVA peptide, SIINFEKL, on the surface of B16-F10 cells. After interferon-gamma (IFN-γ) treatment, B16-F10 cells express high levels of MHC-I molecules on their surface [32, 33], making these cells suitable to study MHC-I-dependent antigen presentation (Fig. 1). Binding of the SIINFEKL peptide to H-2 Kb molecules was monitored by flow cytometry using the antibody 25-D1.16, which specifically recognizes SIINFEKL only when bound to H-2 Kb. As a proof-of-concept that the assay is working, we incubated IFN-γ stimulated B16-F10 cells with the SIINFEKL peptide and determined the presence of the SIINFEKL/H-2 Kb complex at the cell surface (Fig. 1).

### Identification of the optimal spacer for MHC-I-dependent epitope presentation

**Fig. 2:**
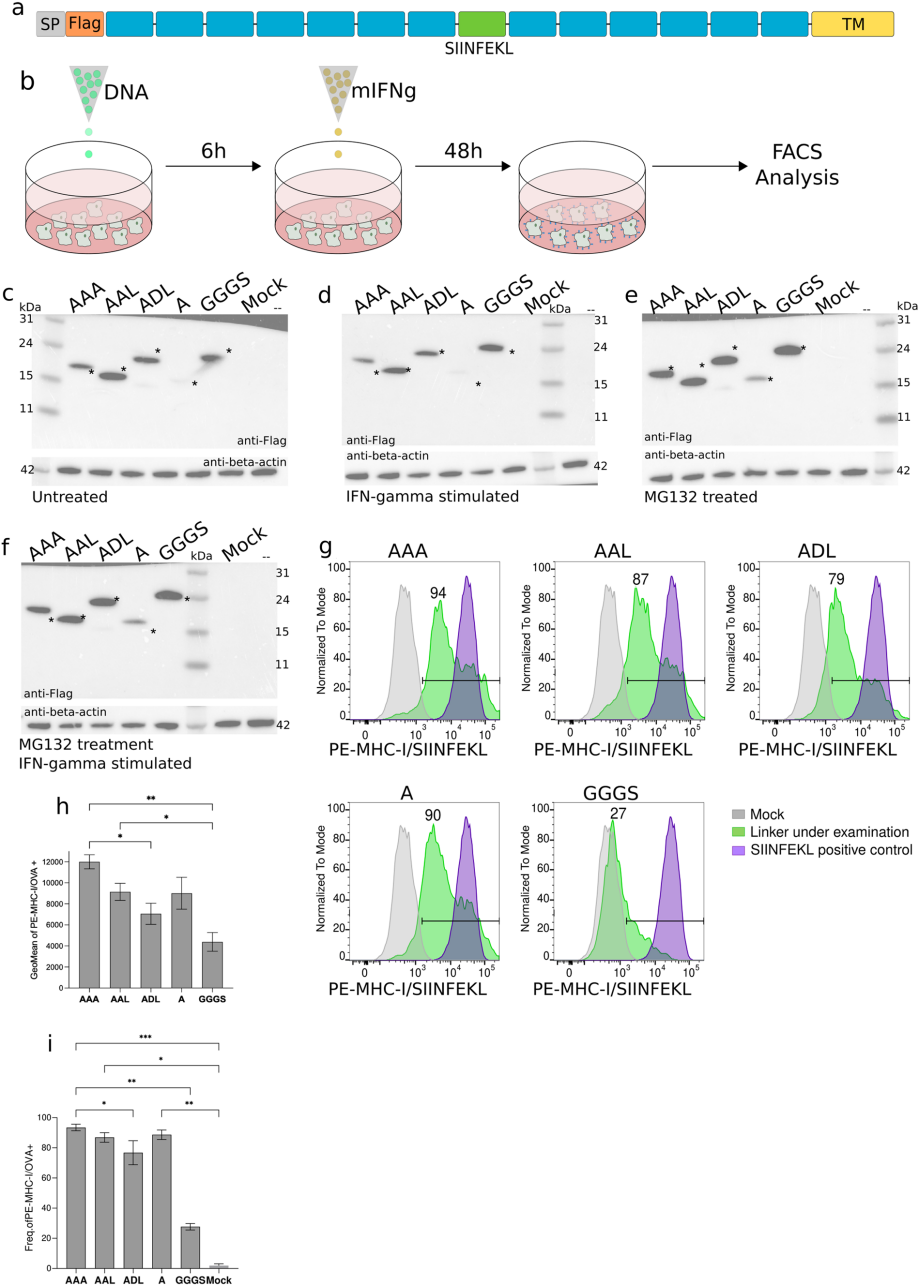
Linker sequences module antigen presentation. **a** Schematic representation of the constructs used. Gray: signal peptide (SP); orange: FLAG tag; blue: neoantigens; green: SIINFEKL peptide; and, yellow: mouse CD44 transmembrane domain. **b** Graphical scheme of the experimental procedure used for linker screening. **c** Western blot analysis of the expression of recombinant proteins untreated transfected B16-F10 cells. Asterisk indicates expected molecular weight. **d** Western blot analysis of the expression of recombinant proteins in IFN-g stimulated transfected B16-F10 cells. Asterisk indicates expected molecular weight. **e** Western blot analysis of the expression of recombinant proteins in MG132-treated transfected B16-F10 cells. Asterisk indicates expected molecular weight. **f** Western blot analysis of the expression of recombinant proteins in IFN-g stimulated and MG132-treated transfected B16-F10 cells. Asterisk indicates expected molecular weight. **g** Representative flow cytometry panels for the detection of MHC-I/SIINFEKL on the surface of transfected B16-F10 cells. Frequency of positive cells for the linker under examination is represented on each panel. Gray: mock; green: linker under examination; purple: SIINFEKL positive control. **h** Levels of MHC-I/SIINFEKL on the surface of B16-F10 transfected cells expressed as geometric mean plus SD of three independent experiments. **i** Frequency of MHC-I/SIINFEKL positive cells. Mean plus SD of three independent experiments is shown. Data were analyzed using Dunn’s test, ^**^P < 0.01, ^*^P < 0.1

To investigate the impact of spacer on the MHC-I presentation of neoantigens included in a polypeptide and select the optimal spacer for a neoantigen polypeptide vaccine, we took advantage of our epitope presentation in vitro assay. We designed a DNA plasmid encoding a single-chain polypeptide containing a signal peptide and the FLAG tag at the N-terminus, followed by a total of thirteen putative B16-F10-specific MHC-I-restricted peptides in addition to the SIINFEKL peptide at the center (Fig. 2a). We then tested five different spacer sequences (AAA, AAL, ADL, A and GGGS). Of note, peptides in each construct were linked by the same spacer. The selection of the spacer sequence was based on cleavage preferences by the immunoproteasome and ERAP proteins (16,19,20,34–40) and on previously published work [22, 26–28].

All five plasmids, coding for surface expressed polypeptides, were separately transfected into B16-F10 cells. Six hours later, cells were treated with IFN-γ to enhance both the expression of MHC-I molecules on the cell surface and the function of the immunoproteasome. Forty-eight hours later, the complex formation between the processed SIINFEKL peptide and MHC-I molecules (H-2 Kb) at the cell surface was determined by flow cytometry [21] (Fig. 2b).

Expression of the full-length proteins was evaluated by western blot using an anti-Flag antibody. Polypeptide molecule containing spacers AAA, AAL, ADL and GGGS were successfully expressed, while A-linked polyprotein was not detected (Fig. 2c), suggesting that it is either not expressed or processed very rapidly. However, stimulation of transiently transfected B16-F10 cells with IFN-γ showed a decreased intensity in the western blot bands for all polyproteins except for GGGS-linked, compared to untreated samples (Fig. 2d). In contrast, incubation of transiently transfected B16-F10 cells with MG132, a proteasomal inhibitor, showed an increased intensity in the western blot bands for all polyproteins, including the A-linked polyprotein, which becomes clearly detectable at its expected molecular weight (Fig. 2e). At last, incubation of transiently transfected B16-F10 cells with MG132 allowed the increase or recovery of all polyprotein expression after IFN-γ stimulation (Fig. 2f). These results suggest that proteasomal degradation may be a major contributor to the processing of the polypeptides.

**Fig. 3:**
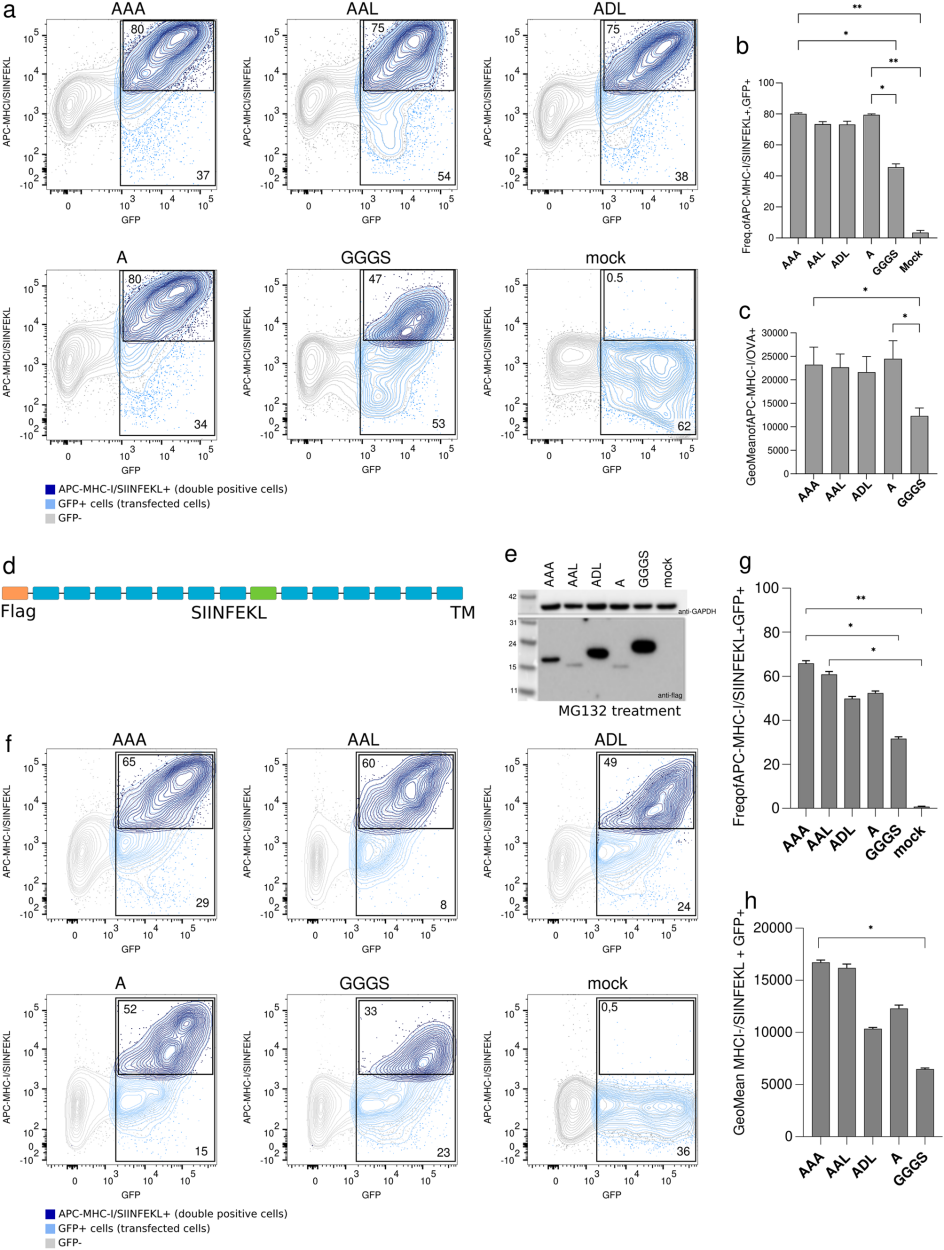
Recombinant proteins transfection controls, surface expressed or intracellularly expressed polypeptides. **a** Representative flow cytometry panels for the detection of MHC-I/SIINFEKL on co-transfected B16-F10 cells with both surface expressed polypeptide and GFP coding plasmid. Frequency of GFP^+^ and APC-MHC-I/SIINFEKL^+^ in GFP^+^ cells are indicated in each panel. Gray: GFP negative cells; green: GFP^+^ cells; red: APC-MHC-I/SIINFEKL^+^ in GFP^+^ cells. **b** Frequency of APC-MHC-I/SIINFEKL^+^ in GFP^+^ co-transfected cells. **c** Levels of detection of MHC-I/SIINFEKL on the surface of GFP^+^ B16-F10 co-transfected cells are showed as geometric mean. **d** Schematic representation of the intracellularly expressed polyproteins used. Orange: FLAG tag; blue: neoantigens; and, green: SIINFEKL peptide. **e** Western blot image of the expression of recombinant intracellularly expressed polypeptides in MG132-treated transfected B16-F10 cells. **f** Representative flow cytometry panels for the detection of MHC-I/SIINFEKL on co-transfected B16-F10 cells with both intracellularly expressed polypeptide and GFP coding plasmid. Frequency of GFP^+^ and APC-MHC-I/SIINFEKL^+^ in GFP^+^ cells are indicated in each panel. Gray: GFP negative cells; green: GFP^+^ cells; red: APC-MHC-I/SIINFEKL^+^ in GFP^+^ cells. **g** Frequency of APC-MHC-I/SIINFEKL^+^ in GFP^+^ co-transfected cells. **h** Levels of detection of MHC-I/SIINFEKL on the surface of GFP^+^ B16-F10 co-transfected cells are showed as geometric mean. Error bars represent SD of three independent experiments. All data were analyzed using Kruskal–Wallis test corrected for multiple comparisons by original FDR method of Benjamini and Hochberg, ^*^P < 0.1, ^**^P < 0.01

Next, the antigen presentation efficiency was determined by the presence of MHC-I/SIINFEKL complexes on the surface of transfected and IFN-γ stimulated B16-F10 cells. Flow cytometry data showed that the highest amount of the MHC-I/SIINFEKL complex was obtained with the AAA-spacer, indicating that this linker might be the most successful spacer for the processing and MHC-I presentation of the SIINFEKL peptide (Fig. 2g). Conversely, the GGGS linker showed the lowest signal indicating that it was less efficient in peptide processing and presentation (Fig. 2g and h). Analysis of the frequencies of cells expressing MHC-I/SIINFEKL complexes confirmed the better presentation and processing of the peptides by alanine-based linkers versus GGGS (Fig. 2i). Such differences were not due to transfection efficiency variations since that was similar in GGGS and AAL-transfected cells (52.7% vs 53.5% of GFP^+^ cells, respectively) and higher than in those cells transfected with AAA, ADL or A polypeptides (37.1%; 38% and 33.9% of GFP^+^ cells, respectively) (Fig. 3a). Analysis of the frequencies of cells expressing MHC-I/SIINFEKL complexes, as well as their signal intensity on the surface of GFP^+^ cells, confirmed the more efficient presentation of the SIINFEKL peptide when neoantigens are concatenated using alanine-based linkers (Fig. 3b and c).

Surface expressed proteins may require extra processing and transport steps, compared to intracellularly expressed proteins, and this fact may affect to protein availability for proteasomal processing and epitope presentation. Therefore, we confirmed the results described above using intracellularly expressed polypeptides (Fig. 3d). Thus, we removed the signal peptide and the CD44-TM of the initial constructs and evaluated their expression in MG132-treated B16-F10-transfected and IFN-γ stimulated cells by western blot. Polypeptides containing spacers AAA, ADL and GGGS were successfully expressed, while AAL- and A-linked constructs showed a weaker signal (Fig. 3e), suggesting that they were either less expressed or processed very rapidly. Then, the antigen presentation efficiency was determined as previously described, including co-transfection with a GFP coding plasmid for transfection efficiency evaluation. AAA-linked polypeptide showed the highest amount of the MHC-I/SIINFEKL complex by flow cytometry, while GGGS-linked polypeptide showed the lowest signal (Fig. 3f-h). These results are in line with those previously obtained with surface expressed polyproteins. Analysis of the frequencies of cells expressing MHC-I/SIINFEKL complexes confirmed a more efficient presentation and processing of the peptides concatenated by alanine-based linkers versus GGGS (Fig. 3g and h).

**Fig. 4:**
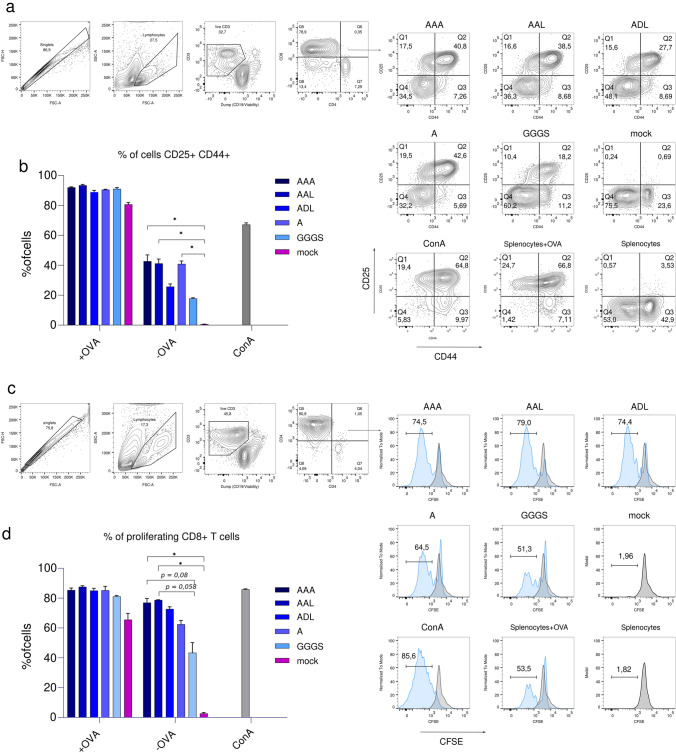
Effect of linker sequence in activation and proliferation of T cells. **a** Gating strategy for the analysis of CD8^+^ T cells activation. **b** Frequency of CD8^+^ CD25^+^ CD44^+^ T cells, 24-h post-co-culture of B16-F10 transfected cells with splenocytes from OT-I mice. **c** Gating strategy for the analysis of the CD8^+^ T cells proliferation. **d** Frequency of proliferating CD8^+^ T cells at 72-h post-co-culture of B16-F10 transfected cells with splenocytes from OT-I mice. Mean plus SD of three replicates is shown in all graphs. All data were analyzed using Kruskal–Wallis test corrected for multiple comparisons by original FDR method of Benjamini and Hochberg, ^*^P < 0.1, ^**^P < 0.01

To confirm that the detection of MHC-I/SIINFEKL complexes on the surface of B16-F10 cells can stimulate CD8^+^ T cells, we performed an antigen presentation and stimulation experiment using splenocytes from OT-I mice. This mouse model expresses a transgenic TCR that recognizes the SIINFEKL peptide in the context of H2Kb [34]. With this aim, B16-F10 cells were transfected with surface expressed polypeptides and stimulated with IFNγ to promote protein processing and presentation. Then, transfected and stimulated B16-F10 cells were co-culture with splenocytes from OT-I mice. Activation of CD8^+^ T cells was evaluated 24-h post-co-culture. The results showed higher frequency of CD25^+^ CD44^+^ CD8^+^ T cells in those co-cultures where B16-F10 cells were transfected with alanine-based linkers (Fig. 4a and b). Similar results were obtained when the proliferating activity of CD8^+^ T cells was evaluated on day three (Fig. 4c and d). Taken together, these results confirm that alanine-based linkers promote a more efficient processing and presentation of the peptides, generating a higher activation of CD8^+^ T cells.

### Effect of neoantigen position on epitope presentation

**Fig. 5:**
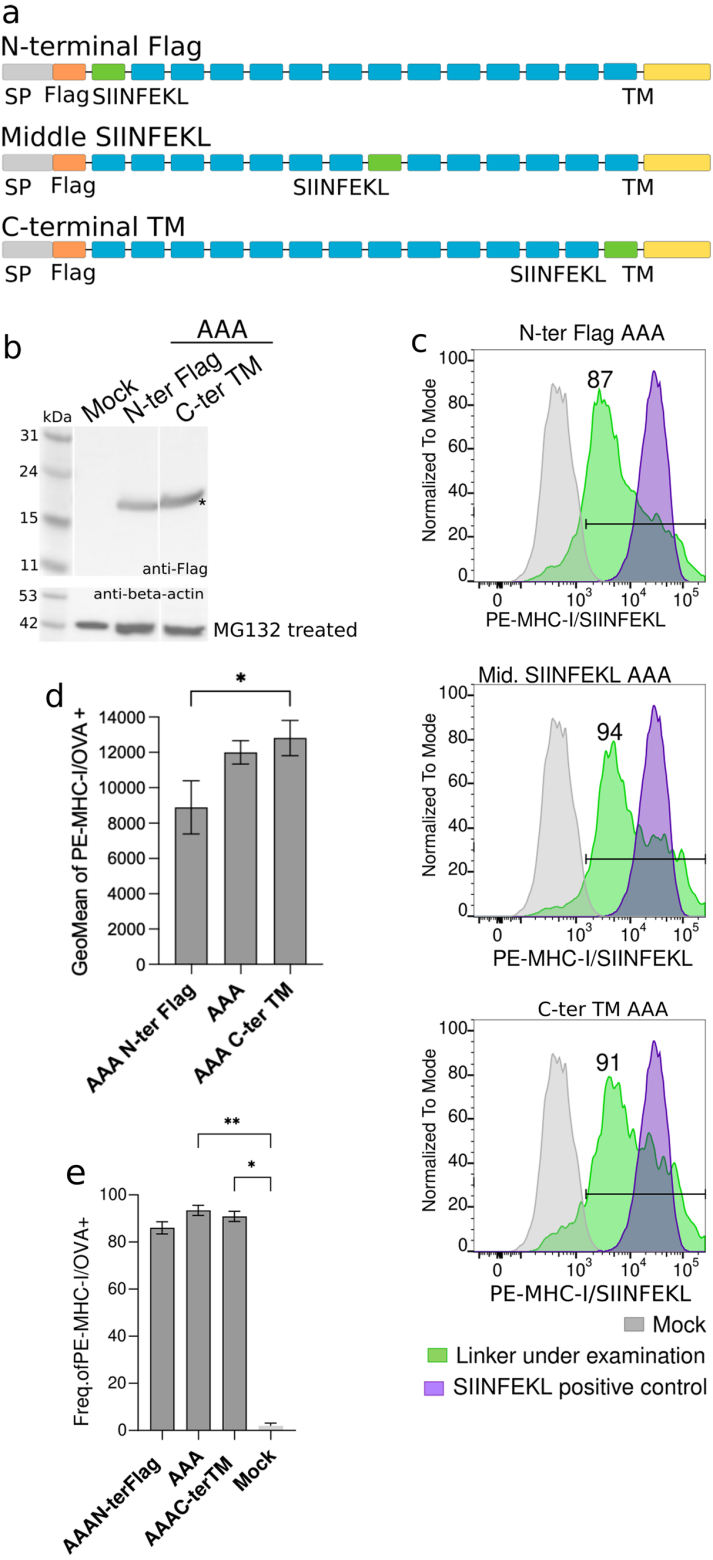
Role of peptide position in antigen presentation. **a** Schematic representation of the constructs used. Gray: signal peptide; orange: Flag tag; blue: neoantigens; green: SIINFEKL peptide; and, yellow: mouse CD44 transmembrane domain. **b** Western blot image of the expression of recombinant proteins in MG132-treated transfected B16-F10 cells. Asterisk indicates expected molecular weight. **c** Representative flow cytometry panels for the presentation of MHC-I/SIINFEKL peptide on transfected B16-F10 cells. Frequency of positive cells is represented on each panel. Gray: mock; green: linker under examination; purple: SIINFEKL positive control. **d** Geometric mean values for the presentation of SIINFEKL peptide on mouse MHC-I molecules from transfected B16-F10 cells with AAA N-term, AAA and AAA C-term TM. Error bars represent SD of three independent experiments. Data were analyzed using Dunn’s test, ^*^P < 0.1

Another open question is whether peptide position within the long polypeptide might affect peptide processing and presentation. To investigate this possibility, three recombinant proteins were tested, where the SIINFEKL peptide was located at the N-terminal region, at the center or close to the C-terminus of the polypeptide sequence (Fig. 5a). All three constructs were designed using the AAA-spacer, since it was the most favorable linker. All resulting proteins were expressed at the expected molecular weight by transient transfection in B16-F10 cells (Figs. 2c and 5b), and the level of presentation of the SIINFEKL peptide located at the different positions was assessed by flow cytometry (Fig. 5c). The SIINFEKL peptide was detected on the surface of the B16-F10 cells in complex with MHC-I molecules in all cases. Similar levels of presentation were observed when the SIINFEKL peptide was expressed at the center or close to the C-terminus of the recombinant protein. However, when expressed at the N-terminal region, the amount of the H2K^b^/SIINFEKL complexes was lower compared to the rest (Fig. 5c-e). These results suggested that processing and presentation of neoantigens at the middle or C-terminus of the polypeptide would be more successful compared to neoantigens at the N-terminal region.

**Fig. 6:**
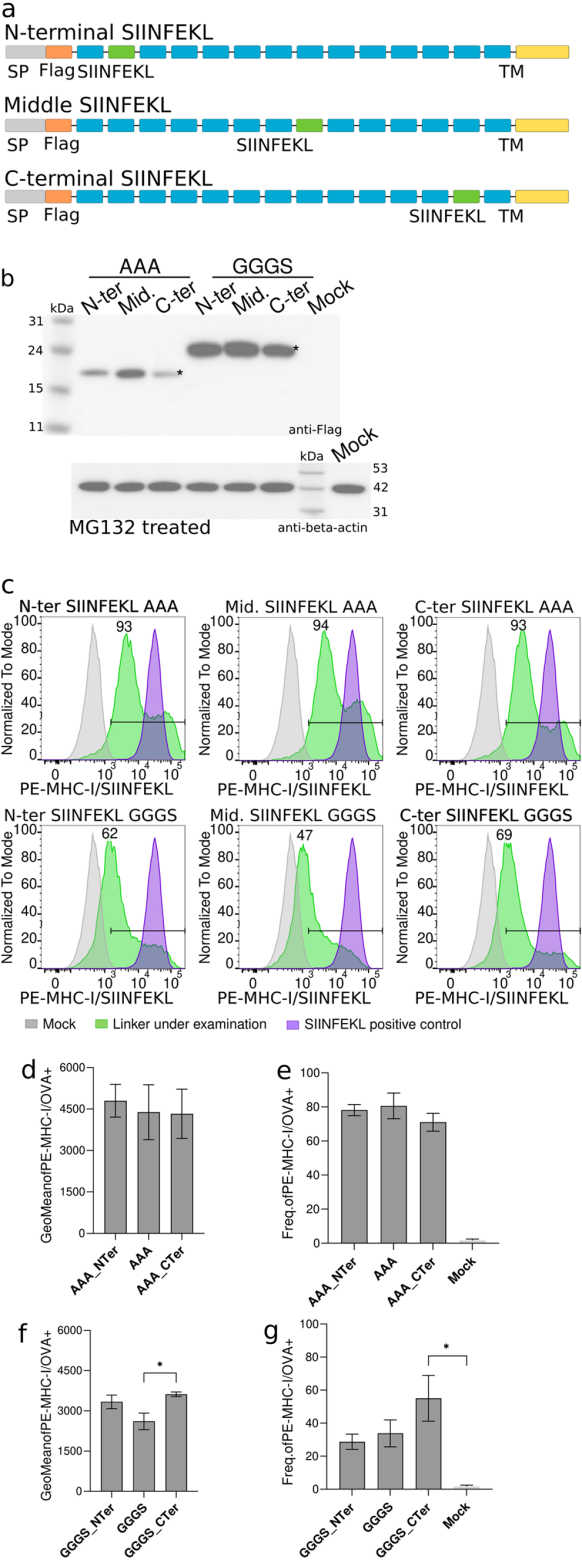
Sequence peptide environment influences processing and MHC-I presentation. **a** Schematic representation of the constructs used. Gray: signal peptide; orange: FLAG tag; blue: neoantigens; green: SIINFEKL peptide; and, yellow: mouse CD44 transmembrane domain. **b** Western blot image of the expression of recombinant proteins in MG132-treated transfected B16-F10 cells. Asterisk indicates expected molecular weight. **c** Representative flow cytometry panels for the presentation of MHC-I/SIINFEKL peptide on transfected B16-F10 cells. Frequency of positive cells is represented on each panel. Gray: mock; green: construct under examination; purple: SIINFEKL positive control. **d** Levels of detection of MHC-I/SIINFEKL on the surface of B16-F10 transfected cells are showed as geometric mean plus SD of three independent experiments. Three different constructs with the SIINFEKL peptide located in the N-terminal, middle or C-terminal regions of the construct were evaluated. All of them used the AAA linker. Data were analyzed using Dunn’s test, ^*^P < 0.1. **e** Frequency of positive cells expressing MHC-I/SIINFEKL complexes at the surface of B16-F10 cells transfected with AAA N-term, AAA and AAA C-term. Mean plus SD of three independent experiments is shown. **f** Levels of detection of MHC-I/SIINFEKL on the surface of B16-F10 transfected cells are showed as geometric mean plus SD of three independent experiments. Three different constructs with the SIINFEKL peptide located in the N-terminal, middle or C-terminal regions of the construct were evaluated. All these constructs used the GGGS linker **g** Frequency of positive cells expressing MHC-I/SIINFEKL complexes at the surface of B16-F10 cells transfected with GGGS N-term, GGGS and GGGS C-term. Data were analyzed using Dunn’s test, ^*^P < 0.1

Alternatively, SIINFEKL presentation might depend on the surrounding amino acids. Since in the previous experiment, the SIINFEKL peptide located at the N-terminal region was flanked by the Flag Tag (DYKDDDDK) and an AAA-linked neoantigen, we designed a new polypeptide where the N-terminal located SIINFEKL peptide was flanked by two neoantigens spaced by the AAA linker or the GGGS (Fig. 6a). In addition, a polypeptide sequence positioning the SIINFEKL peptide at the C-terminal region and spaced by one neoantigen from the transmembrane domain of CD44 was also analyzed (Fig. 6a). Expression of all new AAA- and GGGS-linked recombinant proteins was confirmed by western blot, showing the expected molecular weight for all variants (Fig. 6b). Remarkably, the detection of H2K^b^/SIINFEKL complexes in transfected B16-F10 cells showed no differences among constructs (Fig. 6c–g). Overall, the data suggest that the efficiency of neoantigen processing and presentation might depend on the environment of the flanking sequences (beyond the linker itself), but their position (N-, middle or C-terminal) does not play a major role. Moreover, these results confirm that position of the neoantigen in the polypeptide sequences would not influence the processing and presentation of the neoantigens by the MHC-I, having all the neoantigens the same chance to be represented at the surface of the cells by MHC-I proteins.

## Discussion

Nowadays, the advances in next-generation sequencing (NGS) techniques and the development of new bioinformatic pipelines for the identification and selection of neoantigens expressed by cancer cells have opened the gateway to the use of novel neoantigen-based vaccines as promising therapeutic approaches to fight cancer [35]. For such immunotherapies, neoantigens may be administered as individual peptides or as a single-chain polypeptide protein. Although both strategies have shown encouraging results in clinical trials [4, 5, 7–9], it is thought that a polypeptide chain including multiple neoantigens would increase the efficiency of neoantigens as vaccines [6]. This is especially relevant in the case of RNA- or DNA-based vaccines, since in such formulations, neoantigens are necessarily concatenated in a single molecule. However, there is no clear consensus in the field about the optimal length of the peptides, neither about the spacer sequences that separate concatenated neoantigens in a polypeptide molecule. Regarding the peptides’ length, the trend has been to use LPs which are able to generate CD8^+^ and CD4^+^ T cell-mediated immune responses [11, 14, 15]. However, in order to specifically increase the activation of CD8^+^ T cell responses, LPs need to be processed to 8–11 amino acids length, in order to be properly presented by MHC-I molecules. It is well-known that flanking sequences have a major impact on peptide processing and MHC-I presentation [36, 37]. In this way, Velders and colleagues [24] demonstrated that the addition of a linker sequence flanking different antigens included in a single DNA molecule improved their MHC-I presentation and in vivo protection in a mouse cancer model. A diverse set of linker sequences have been investigated including short and flexible spacers rich in glycine and serine [25–27, 29] as well as protease cleavage site, such as furin [23]. Special emphasis has been made on the use of short linkers rich in hydrophobic amino acids (alanine, leucine or tyrosine) [6, 22, 24, 25, 28, 31], which are the preferred cleavage sites of the immunoproteasome and the ERAP1/2 machinery [16, 19, 20].

In order to bring some light on these issues, we have used a flow cytometry-based method to test which linker would allow the optimal processing and presentation of neoantigens present in a polypeptide molecule. This method is based on the detection of the OVA peptide SIINFEKL, as a reporter epitope, bound to MHC-I molecules using the antibody 25-D1.16, which specifically recognizes the MHC-I/SIINFEKL complex. In addition, we confirmed the cytometry results using a functional in vitro assay based on the stimulation of the SIINFEKL-specific CD8^+^ T cells (OT-I CD8^+^ T cells). Therefore, the cytometry method used here is a good alternative to mass spectrometry, or in vivo and in vitro cell-based antigen presentation assays, which are more expensive, time-consuming or need the use of animal models. According to our results, the optimal spacer, among those that have been tested, is a triple alanine linker (AAA). In contrast, a very common and more flexible spacer containing three glycine and one serine (GGGS) has shown the poorest results. Our data suggest that linkers based on alanine residues would allow more efficient processing and presentation of the neoantigens than linkers including combinations of glycine and serine. The inclusion of alanine in linkers is not new. Structured linkers containing alanine [(AP)n or (EAAAK)n] have been used in some recombinant fusion proteins [38]. Moreover, they have been also reported in polypeptide vaccines for infectious disease or cancer [24, 28]. However, from our knowledge, this is the first time that the advantage of using alanine-containing linkers compared to GS linkers has been documented in the context of MHC-I antigen presentation.

Once the optimal spacer was selected, we evaluated the effect of the position of the peptide and flanking sequence within the polypeptide molecule on the efficiency of peptide processing and presentation. Initially, our results showed that despite the peptide was processed and presented regardless its position in the polypeptide, its processing/presentation was reduced when located at the N-terminal portion of the polypeptide. On the contrary, peptides from the center to the C-terminus of the molecule were presented to a similar higher level. However, re-arrangement of the peptides at the N-terminus of the molecule showed that the amino acid environment surrounding the peptide might be more relevant than the position in the polypeptide sequence for peptide processing and presentation through the MHC-I pathway. This information should be taken into consideration when a neoantigen-based polypeptide is designed or its immunogenicity evaluated.

Although our results show that alanine-containing linkers improve MHC-I presentation of the flanked peptide, it is still possible that novel MHC-I binding peptides containing the whole or partial linker sequence are generated. These peptides might eventually generate novel T cell specificities that can impact on the global vaccine-induced T cell responses. This impact, regarding immunodominance, clonality and functionality, needs to be evaluated in detail in order to definitely assess the advantage of using alanine-containing peptides, particularly the AAA one, in the generation of polypeptide vaccines based on MHC-I-neoantigens. In addition, since APCs play a major role in in vivo antigen presentation, which in turn depend on several factors (i.e., type of APC, cytokine milieu, or co-stimulation), to confirm our results using different APCs in several physiological stimulation contexts will be also very informative.

In summary, we have shown here that both linker sequence and peptide environment play a major role in the processing and presentation of the epitopes included in a polypeptide molecule. Alanine-based linkers, particularly the AAA-spacer, improved neoantigen presentation when compared with the GGGS linker. Therefore, both aspects, linkers and sequence environment, will likely modulate the immune response generated against each neoantigen in personalized polypeptide vaccines.

### Supplementary Information

Below is the link to the electronic supplementary material.

Supplementary file1 (TIFF 303 KB)
