# Supplementary figures and images for: Alanine-based spacers promote an efficient antigen processing and presentation in neoantigen polypeptide vaccines
Source: Cancer Immunol Immunother. 2023 Feb 23;72(7):2113–25. doi: 10.1007/s00262-023-03409-3 (PMC10264286; doi:10.1007/s00262-023-03409-3)

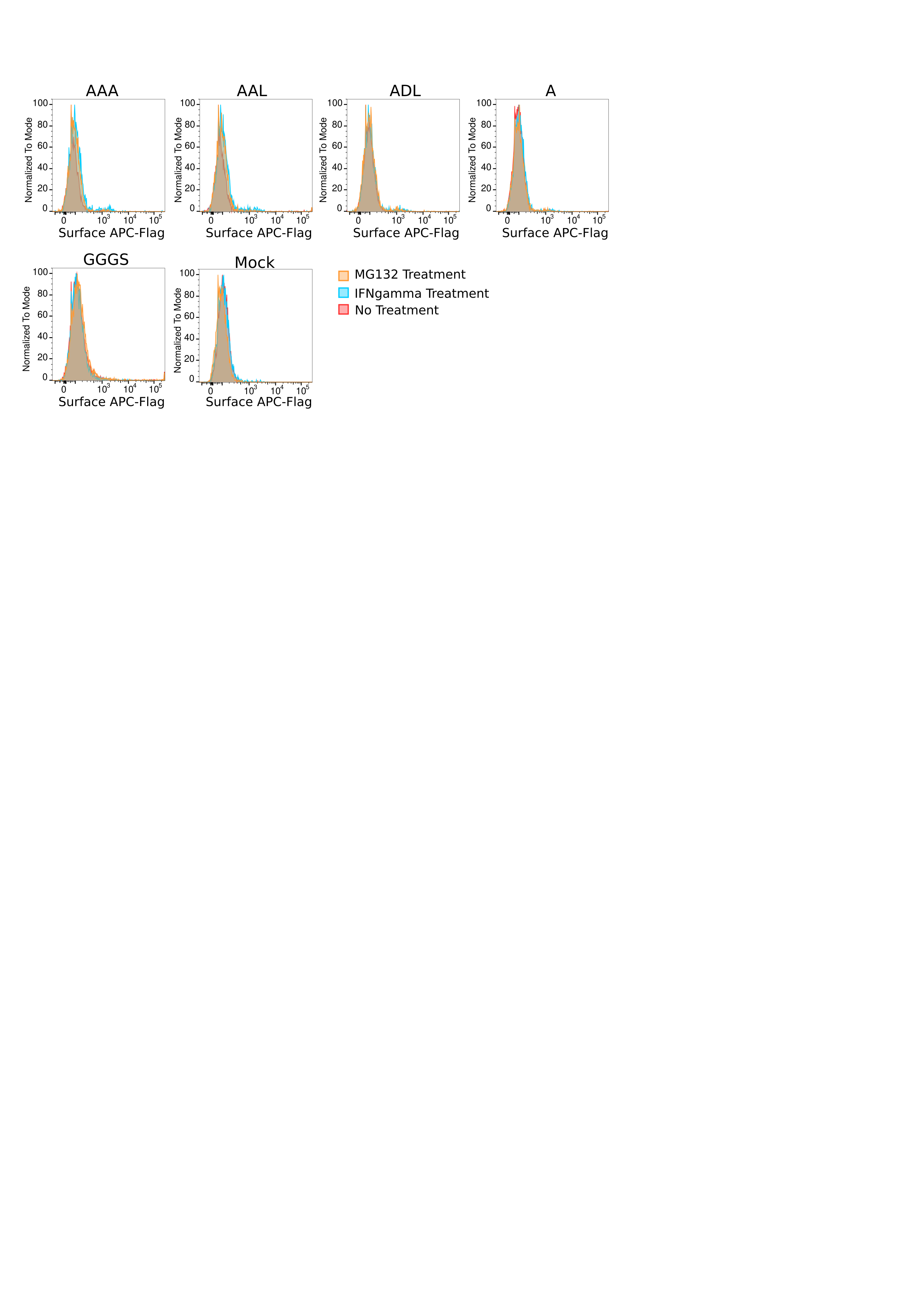

Supplement: Supplementary file 1 — Supplementary file1 (TIFF 303 KB) [file 262_2023_3409_MOESM1_ESM.tiff]
